# PET Imaging of New Target PARP in Prostate Cancer

**DOI:** 10.3390/ph19071020

**Published:** 2026-06-30

**Authors:** Zhao Yang, Wei Wang, Xuanyi Dai, Liya Wei, Yanli Li, Wenfeng Gou, Feifei Xu

**Affiliations:** 1 Department of Molecular Imaging and Nuclear Medicine, Tianjin Medical University Cancer Institute and Hospital, National Clinical Research Center for Cancer, Key Laboratory of Cancer Prevention and Therapy, Tianjin’s Clinical Research Center for China, Tianjin 300060, China; yangzhao@tjmuch.com; 2 State Key Laboratory of Advanced Medical Materials and Devices, Tianjin Key Laboratory of Radiation Medicine and Molecular Nuclear Medicine, Tianjin Institutes of Health Science, Institute of Radiation Medicine, Chinese Academy of Medical Sciences & Peking Union Medical College, Tianjin 300192, China; ww1091709167@yeah.net (W.W.); s2023016021@pumc.edu.cn (L.W.); liyanli@irm-cams.ac.cn (Y.L.); 3 Tianjin Farragut School, Tianjin 300074, China; 18512207235@163.com

**Keywords:** prostate cancer, PARP, radiotracer, [^68^Ga]Ga-FL9-7, PET/CT imaging

## Abstract

**Background/Objectives**: Poly (ADP-ribose) polymerase (PARP), particularly PARP-1, is overexpressed in prostate cancer and linked to poor prognosis. PARP inhibitors show efficacy in homologous recombination deficiency (HRD)-positive tumors, but 30–70% of patients develop resistance, often due to low PARP expression. Tissue biopsies have limitations in assessing PARP levels, highlighting the need for noninvasive imaging tools. This study aimed to develop a novel [^68^Ga]Ga-PARP-targeted radiotracer for prostate cancer visualization and therapy monitoring, with potential implications for targeted radionuclide therapy. **Methods**: PET/CT imaging was conducted in 22RV1 prostate cancer xenograft-bearing mice using the [^68^Ga]Ga-FL9-7 probe. Imaging was performed at 1, 2, and 3 h post-injection. Standardized uptake values (SUV) were quantified to evaluate tumor and organ uptake, and tumor-to-normal tissue (T/NT) contrast ratios were calculated. **Results**: [^68^Ga]Ga-FL9-7 rapidly accumulated in tumors, with optimal imaging contrast achieved at 3 h post-injection. Normal organ uptake (e.g., kidneys) peaked at 1 h and subsequently declined, while tumor uptake increased over time. This differential clearance and retention resulted in the highest T/NT ratio at the 3 h time point. **Conclusions**: The [^68^Ga]Ga-FL9-7 probe enables effective noninvasive visualization of PARP-1 expression in prostate cancer, demonstrating clinical potential for tumor localization and monitoring of PARP-targeted therapies. This work also lays the groundwork for further development of PARP-targeted radionuclide therapy strategies.

## 1. Introduction

Prostate cancer remains a significant global health concern, with incidence and mortality rates varying widely across regions [1,2]. While screening with PSA and DRE has improved early detection, it is not without controversy due to issues of overdiagnosis and overtreatment. Poly (ADP-ribose) polymerase (PARP) is an abundantly expressed enzyme predominantly localized in the nucleus and cytoplasm, where it plays pivotal roles in DNA repair and gene expression regulation [3,4]. Among the PARP family members, PARP-1 has been consistently reported to be overexpressed in various malignancies, including prostate cancer, as evidenced by the Human Protein Atlas database and prior studies. Notably, elevated PARP-1 expression in tumors has been independently correlated with poor patient prognosis, underscoring its clinical significance [5,6,7]. In tumors with homologous recombination deficiency (HRD), PARP inhibitors exert their cytotoxic effects by inducing transcription-replication conflicts through PARP-1 inhibition, ultimately leading to DNA damage accumulation and tumor cell apoptosis [4,8,9].

PARP inhibitors have been extensively investigated across multiple cancer types, demonstrating promising antitumor efficacy [10,11,12]. While PARP inhibitors have demonstrated promising antitumor efficacy across multiple cancer types and have been approved by the FDA for treating Breast Cancer Susceptibility Gene (BRCA)-mutated breast cancer and advanced ovarian cancer, their therapeutic response is not universal. Approximately 30–70% of HRD patients exhibit poor response to PARP inhibitors, with emerging evidence suggesting that low PARP expression in tumor cells may contribute to this resistance [13,14,15,16]. This highlights the critical need for accurate assessment of PARP expression to optimize clinical outcomes.

PSMA ligands are effective for imaging and therapy of prostate cancer, but they target prostate-specific membrane antigen, which may be downregulated in certain aggressive or neuroendocrine-differentiated tumors. In contrast, PARP-1 is overexpressed in a broader range of prostate cancer subtypes, including those with homologous recombination deficiency (HRD), and its expression level correlates with response to PARP inhibitors. Our ^68^Ga-FL9-7 probe specifically addresses the need for non-invasive assessment of PARP-1 expression, which cannot be achieved by PSMA imaging. Given the challenges posed by tumor heterogeneity and the limitations of invasive tissue biopsies in evaluating whole-body PARP expression, noninvasive nuclear medicine imaging using PARP-targeted radiotracers has emerged as a valuable tool for comprehensive lesion assessment. Several [^68^Ga]Ga-PARP-targeted agents, developed based on PARP inhibitor scaffolds, have shown excellent performance in cancer imaging [17,18,19,20,21].

However, the clinical utility of PARP inhibitors remains primarily limited to tumors with BRCA mutations or HRD. The relatively low prevalence of BRCA mutations (approximately 5% in breast cancer and 15% in ovarian cancer) significantly restricts their broader application [12,22,23,24]. To address this limitation, we leveraged the frequent overexpression of PARP in malignancies to develop a novel ^68^Ga radiodiagnostic agent. This agent was designed based on the olaparib-PARP-1 interaction model and demonstrated exceptional tumor targeting and significant antitumor efficacy in PARP-1-positive prostate cancer xenograft models through comprehensive in vitro and in vivo evaluations. To our knowledge, this work represents the first successful development of a Ga-68-labeled PARP-targeted radiodiagnostic agent based on the olaparib scaffold that demonstrates both high tumor uptake and favorable clearance kinetics in a prostate cancer xenograft model, complementing existing PARP tracers reported in the literature, opening new avenues for precision oncology.

## 2. Results

### 2.1. Investigation of FL9-7’s Inhibitory Activity and Targeting Effect on PARP1 via Western Blotting in 22RV1 Cells

**Figure 1:**
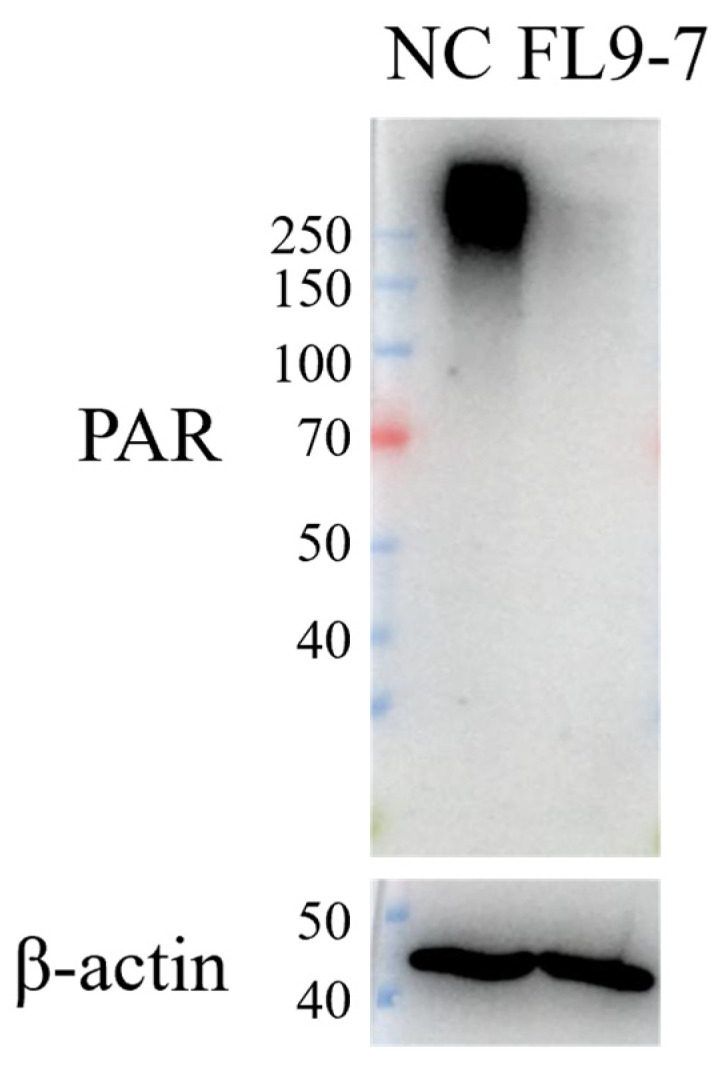
Representative Western blot image in 22RV1 cells treated without or with FL9-7. β-actin serves as a loading control.

To investigate the inhibitory activity and specific targeting effect of FL9-7 on PARP1, we conducted Western blotting experiments. Herein, 22RV1 cells, a well-characterized cell line relevant to the research context, were selected as the experimental model. Through a Western blotting experiment, we found that the activity of PARP1 was significantly inhibited after the addition of FL9-7 to 22RV1 cells (Figure 1). This result indicates that FL9-7 has a potential inhibitory effect on PARP1 in 22RV1 cells, which provides important clues for further exploring the mechanism of action of FL9-7 and its potential application in relevant diseases related to PARP1 over-activity.

### 2.2. Molecular Docking Analysis of FL9-7 with PARP-1

**Figure 2:**
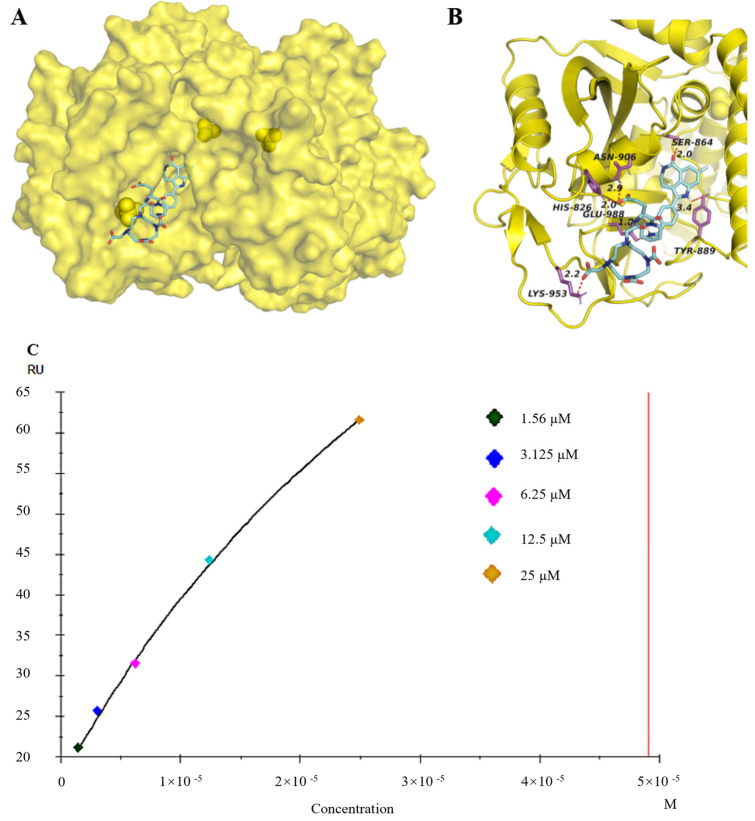
Predicted binding model of FL9-7 with PARP-1 protein. The structure of FL9-7 is colored blue, and the backbone of PARP-1 protein is displayed in the form of surface (**A**) and cartoon (**B**), which is colored yellow by elements in PyMOL. The hydrogen bonds are indicated as dashed lines. (**C**) FL9-7 binds PARP-1 with micromolar affinity, as determined by surface plasmon resonance (SPR). Linear regression fitting of SPR response units (RU) plotted against serial FL9-7 concentrations. Colored diamond data points represent FL9-7 concentrations spanning 1.56 μM to 25 μM; the solid black line denotes the linear regression fit. The vertical red line indicates the upper concentration limit (5 × 10^−5^ M) on the x-axis.

Molecular docking serves as a powerful tool in drug design and structure-activity analysis. It enables the prediction of the binding modes between our synthetic ligands and PARP-1. Considering the recognizability of Schrodinger software, precursor compounds FL9-7 were used for flexible docking (Figure 2A,B). First and foremost, the ligand can fully penetrate the cavity of PARP-1 protein and effectively bind to the key amino acids SER864, thereby exerting its function. Moreover, the DOTA structure, due to its larger size and charged carboxylic acid, ultimately extends like a “tail” outside the binding pocket, forming hydrogen bond interactions with residue LYS953, which further enhances the interaction and overall stability between the ligand and protein. Surface plasmon resonance (SPR) analysis revealed that FL9-7 binds to the catalytic domain of PARP-1 with a dissociation constant of 4.908 × 10^−5^ M (Figure 2C). This affinity falls within the micromolar range, indicating moderate binding strength under the experimental conditions employed.

### 2.3. PET/CT Imaging in Tumor-Bearing Mice

**Figure 3:**
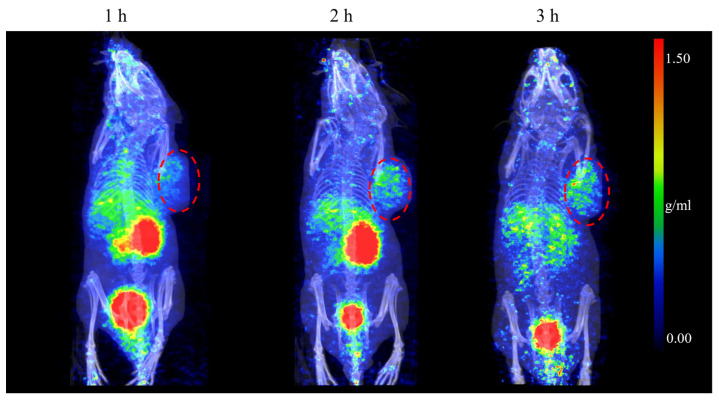
Time-dependent PET/CT imaging of [^68^Ga]Ga-FL9-7 in 22RV1 tumor-bearing mice (*n* = 3). Red circles indicate the region of interest for monitoring dynamic probe enrichment in the focal tissue over time.

To assess the ability of probe [^68^Ga]Ga-FL9-7 to bind PARP-1 in vivo and its biodistribution, we performed PET/CT imaging in 22RV1 prostate cancer tumor-bearing mice to reveal its pharmacokinetic profile in vivo. Figure 3 shows the imaging results of probe [^68^Ga]Ga-FL9-7 at 1 h, 2 h and 3 h. PET/CT images of live tumor-bearing mice showed that [^68^Ga]Ga-FL9-7 was rapidly enriched at the tumor site, making the tumor location clearly visible (marked by red dotted line). Images from 1 h of injection showed significant uptake of [^68^Ga]Ga-FL9-7 at the tumor site, but there was also significant uptake in normal organs and blood pools, resulting in low image contrast. With the passage of time, images injected for 2–3 h showed a significant increase in the metabolism of [^68^Ga]Ga-FL9-7 in normal organs, while the uptake at the tumor site gradually increased. The 2 h image could clearly display the location and size of the tumor, while the 3 h imaging achieved the best results, with the highest image contrast.

**Figure 4:**
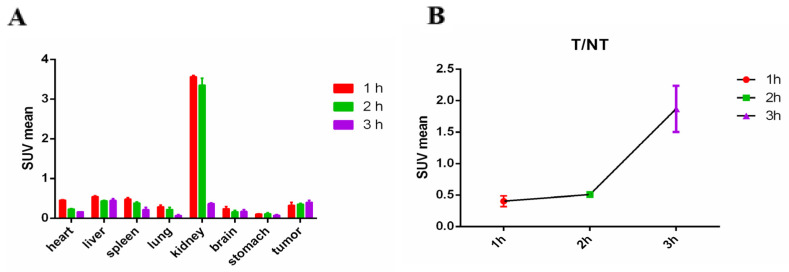
PET Imaging in Tumor-Bearing Mice. (**A**) SUV mean values of probes in various organs and tumors. (**B**) The SUV mean ratio (T/NT) of [^68^Ga]Ga-FL9-7 in tumor tissue and major non-target organs.

The Standardized Uptake Value (SUV) is a commonly used semi-quantitative indicator in tumor diagnosis, which refers to the ratio of the radioactive activity of imaging agents absorbed by local tissues to the average injection activity throughout the body. This study used SUV mean values to evaluate the uptake by various organs and tumor tissues in mice. The uptake of probe ^6^[^68^Ga]Ga-FL9-7 in normal organs reaches its maximum value within 1 h, and then gradually decreases with time, especially in the kidneys. In contrast, the uptake in tumor tissue shows a gradually increasing trend and eventually tends to a stable level (Figure 4 and Table S2). In conclusion, [^68^Ga]Ga-FL9-7, a PARP-1-targeted PET probe, demonstrates rapid and specific tumor accumulation with efficient background clearance in 22RV1 prostate cancer-bearing mice, achieving optimal tumor-to-background contrast at 3 h post-injection, significantly outperforming earlier time points and establishing a robust pharmacokinetic foundation for clinical translation.

## 3. Discussion

The [^68^Ga]Ga-FL9-7 probe demonstrates significant technical advantages in PARP-1 imaging for prostate cancer compared to [^18^F]-PARP probes. Its preparation convenience stems from ^68^Ga being eluted from a germanium-gallium generator (^68^Ge–^68^Ga), eliminating reliance on cyclotron production, which enhances clinical flexibility. In contrast, 18F-labeled probes require cyclotrons, limiting their widespread use in non-nuclear medicine centers. Pharmacokinetically, [^68^Ga]Ga-FL9-7 exhibits rapid tumor accumulation and selective clearance, with optimal imaging contrast achieved 3 h post-injection when the tumor-to-background ratio (T/NT) peaks. This time-dependent profile arises from rapid clearance in normal organs (e.g., kidneys) and sustains retention in tumor tissue. Studies show progressive tumor uptake of [^68^Ga]Ga-FL9-7 in prostate cancer xenografts, while normal organ uptake peaks at 1 h and declines, creating ideal conditions for precise tumor localization.

Despite its potential, [^68^Ga]Ga-FL9-7 faces challenges in clinical application. Limited clinical data require larger trials to verify diagnostic efficacy, particularly in correlating PARP-1 expression levels in prostate cancer patients. Approximately 30–70% of HRD patients exhibit primary resistance to PARP inhibitors, often due to low PARP expression [25], and [^68^Ga]Ga-FL9-7 imaging can identify these resistant patients to guide diagnosis adjustments. Regulatory hurdles include extended review under China’s special medical device approval process, though guidelines like Technical Guidelines for Clinical Evaluation of Radioactive Diagnosis Drugs provide reference standards. Cost-effectiveness is another consideration, as demonstrating added clinical value over traditional imaging (e.g., PSMA PET) is essential for reimbursement. China’s “14th Five-Year Plan” includes 860 PET/CT units to support adoption.

Synergistic applications of [^68^Ga]Ga-FL9-7 imaging and PARP inhibitors include diagnostic guidance, where PARP-1 expression correlates with inhibitor efficacy [26,27,28]. Imaging identifies candidates for precision therapy. Treatment monitoring is facilitated by dynamic PARP-1 expression assessment, providing objective efficacy metrics [29]. Early trials show 46% pathological remission with PARP inhibitor-neoadjuvant androgen therapy in high-risk prostate cancer [30]. Patient stratification based on PARP-1 expression (high/low) allows tailored treatments, potentially improving efficacy [31].

Future directions include adapting [^68^Ga]Ga-FL9-7’s scaffold for diagnostic radionuclides (e.g., ^177^Lu) to enable “diagnosis-therapy” integration, supported by China’s Medium-Long-Term Plan for Medical Isotopes. Expanding its utility to other PARP-1-overexpressing cancers (e.g., 50% of ovarian cancer cases) is also promising. AI integration through automated algorithms can quantify PARP-1 expression and enhance diagnostic consistency. Global multicenter trials are planned to validate [^68^Ga]Ga-FL9-7’s efficacy across diverse populations, leveraging China’s existing PET/CT infrastructure.

## 4. Materials and Methods

### 4.1. Chemical Synthesis

**Scheme 1:**
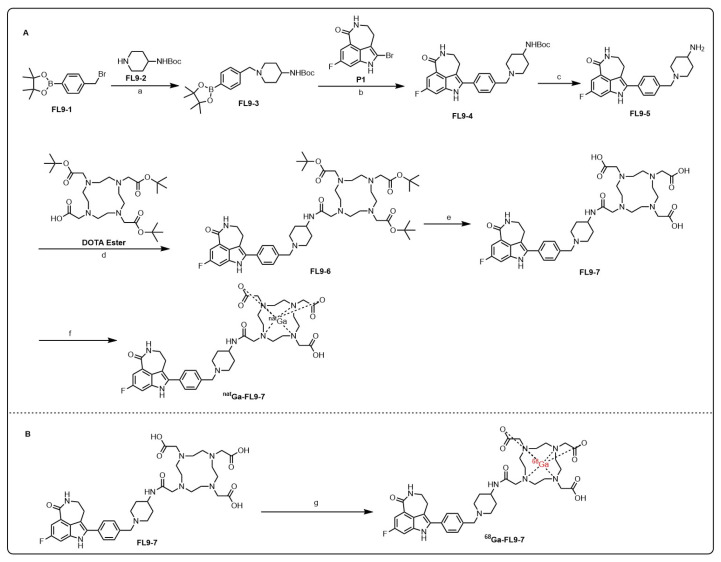
Synthetic Routes and Labeling Methods for the Target Compounds. Reagents and conditions: (**A**) (a) K_2_CO_3_, ACN, r.t., 6 h; (b) Pd(dppf)Cl_2_, Na_2_CO_3_, Dioxane/H_2_O = 6/1, 90 °C, 4 h; (c) TFA/DCM = 1/3, r.t., 1 h; (d) HATU, DIPEA, DMF, r.t., 3 h; (e) TFA/DCM = 2/1, r.t., 12 h. (f) ^nat^GaCl_3_, HCl, NaOAc, 95 °C, 10 min. (**B**) (g) ^68^GaCl_3_, HCl, NaOAc, 95 °C, 10 min.

The synthesis of the target compound was accomplished according to the synthesis routes of Scheme 1. Firstly, using K_2_CO_3_ as base, the starting materials FL9-1 and FL9-2 undergo an SN2 nucleophilic substitution reaction to yield the intermediate compound FL9-3. Compound FL9-3 and the starting material P1 are then subjected to a Suzuki coupling reaction under reflux conditions, using Na_2_CO_3_ as the base, to generate the intermediate compound FL9-4. Subsequently, the Boc protecting group is removed in a TFA/DCM system to afford FL9-5. Next, compound FL9-5 was then subjected to an acid-amine condensation reaction with DOTA Ester at room temperature to produce intermediate compound FL9-6. Finally, the TFA/DCM system was used to hydrolyse the tert-butyl ester group to obtain the precursor compound FL9-7. The nonradioactive gallium complex [natGa]Ga-FL9-7 was synthesized by chelating the precursor FL9-7 with GaCl_3_ in acetate buffer. The resulting products were then purified by RP-HPLC and freeze-dried to achieve a white powder with a purity greater than 98%. Full experimental details can be found in the Experimental Section and Supporting Information. Intermediates and the final product were characterized by proton nuclear magnetic resonance (^1^H NMR) and mass spectrometry (MS) (Figures S1–S9). The purity of FL9-7 is greater than 95% (Figure S10) for subsequent in vivo and in vitro evaluations.

### 4.2. Radiolabeling Experiments

After investigation and optimization of the conditions, including pH, temperature, and reaction time, all of the radioactive compounds with high purity were obtained and directly used for vivo imaging. The [^68^Ga]GaCl_3_ solution was washed from a ^68^Ge–^68^Ga generator (ITM, Garching, Germany) in a volume of 5 mL (in 0.1 MHCl, 1.11 GBq). The pH of the [^68^Ga]GaCl_3_ solution was adjusted to 4.5 using sodium acetate (0.4 M, 0.5 mL) in 0.1 M hydrochloric acid. This resulted in a total reaction mixture volume of approximately 5.5 mL. Subsequently, 20 μg of the FL9-7 precursor (dissolved in 20 μL H_2_O) was added into the mixture, which was then heated at 95 °C for 10 min to obtain the labeling product (Scheme 1B). After the reaction, the mixture was loaded onto a preconditioned C18 solid-phase extraction cartridge (Sep-Pak C18 Plus Light Cartridge Waters Corporation, Milford, MA, USA). The cartridge was washed with 10 mL of saline and 5 mL of ultrapure water to remove free ^68^Ga^3+^ and gallium (Ga^3+^) salts. The radiolabeled product [^68^Ga]Ga-FL9-7 was finally eluted with 1 mL of ethanol and diluted with saline for subsequent experiments. The radiochemical purity of the [^68^Ga]Ga-FL9-7 was confirmed to be >95% by radio-TLC (Figure S12). 

### 4.3. Molecular Docking

The X-ray co-crystal structure of PARP-1 in complex with Rucaparib was obtained from the RCSB Protein Data Bank (PDB ID: 4RV6). Chain A was selected as the receptor model for molecular docking. The receptor was prepared by removing water molecules, adding hydrogen atoms, and retaining the co-crystallized Rucaparib temporarily to define the active binding pocket using the protein preparation module in Glide within the Schrödinger software suite. The docking was performed as target-based docking rather than blind docking. The receptor grid was generated based on the centroid of the co-crystallized Rucaparib ligand in chain A, with the grid center set at approximately X = 57.20, Y = 3.95, and Z = 120.85 Å. The grid box size was set to 14 Å to cover the Rucaparib binding pocket. No additional metal ions were introduced during receptor preparation (Table S1).

The structures of Rucaparib and FL9-7 ligands were drawn using ChemDraw 19.0 and further prepared using LigPrep in Schrödinger 2018-1. After receptor preparation and grid generation, molecular docking was carried out using Glide flexible docking. Other docking parameters were kept as the defaults. The docking results were analyzed by comparing the binding poses, docking scores or Gibbs free binding energy values, hydrogen bonds, and other non-covalent interaction profiles, including hydrophobic interactions, π-related interactions, and electrostatic contacts. The interaction patterns of FL9-7 ligands were compared with the co-crystallized Rucaparib binding mode in 4RV6. The final docking poses and interaction diagrams were visualized using PyMOL (Version 2.6, Schrödinger, LLC, New York, NY, USA).

### 4.4. Surface Plasmon Resonance (SPR) Binding Assays

To investigate the affinity of [^68^Ga]Ga-FL9-7 with PARP-1 protein, we selected their corresponding precursors FL9-7 for the surface plasmon resonance (SPR) binding experiments with PARP-1. The surface plasmon resonance binding assay was performed according to a previous protocol. The PARP-1 protein used in the experiment was a recombinant human protein (HY-P74652), purchased from MCE. The Tris buffer was replaced by passing the sample through a protein column. The sensor chip used was the CM5 model (Cat. No. BR-1005-30, Cytiva, Shanghai, China).

### 4.5. Western Blot Analysis

Prostate cancer cells were harvested and lysed using RIPA buffer (Solarbio, Beijing, China) containing protease inhibitors. Protein concentration was determined using a BCA assay kit (Solarbio, Beijing, China). Subsequently, protein samples underwent denaturation and separation via 8–15% SDS-PAGE before being transferred onto polyvinylidene fluoride (PVDF) membranes (Merck Millipore, Billerica, MA, USA). Following transfer, membranes were blocked with 5% non-fat dry milk in TBST buffer and incubated with primary antibodies at 4 °C overnight. After thorough washing, membranes were exposed to appropriate secondary antibodies for 2 h at room temperature. Protein bands were finally visualized using an enhanced chemiluminescence (ECL) detection system (Millipore, Billerica, MA, USA).

### 4.6. In Vivo Experimental Procedures

The Animal Ethics Committee of the Institute of Radiation Medicine, Chinese Academy of Medical Sciences and Peking Union Medical College approved all experimental procedures (approval number: IRM2-IACUC-2512-022). Specific pathogen-free (SPF) male BALB/c nude mice (4-week-old) were sourced from Beijing Huafukang Biotechnology Co., Ltd. (Beijing, China) and maintained in the SPF animal facility at the Institute of Radiation Medicine (IRM), Chinese Academy of Medical Sciences. 

### 4.7. Tumor Model Establishment

Subcutaneous implantation of 1 × 10^6^ PARP-high 22RV1 cells (suspended in Hank’s balanced salt solution) was performed in the right flank region. Tumor growth was monitored until volumes reached 300–500 mm^3^, at which point animals were allocated for imaging or biodistribution studies. No formal power analysis was conducted. The sample size of *n* = 3 per group was selected based on established precedent in preclinical studies using the 22RV1 xenograft model, where this number consistently provides sufficient statistical power to detect clinically relevant differences in tumor growth under standard imaging protocols. Animals were included if tumor volume reached 300–500 mm^3^ at the time of randomization, as measured by calipers using the formula *V* = (*L* × *W*^2^)/2, where *L* is the longest diameter and *W* is the perpendicular diameter. Exclusion criteria were predefined as: (1) tumor volume < 200 mm^3^ or >600 mm^3^ at allocation; (2) weight loss > 20% from baseline; (3) signs of distress, infection, or ulceration at injection site; (4) failure of tumor engraftment (no palpable mass at day 7 post-inoculation). All exclusions were documented and reported. No animals or data points were excluded in this study.

### 4.8. Imaging Protocol

For PET imaging, mice were anesthetized with 3% (*v*/*v*) isoflurane (maintenance at 1.5%). A standardized dose of 3.7 MBq [^68^Ga]Ga-FL9-7 was administered via tail vein. Dynamic PET/CT scans (IRIS system, Inviscan SAS, Strasbourg, France) were acquired at 1, 2, and 3 h post-injection, each scan duration was 15 min. Image reconstruction and analysis were performed using automated imaging software OsiriX Lite v7.5 (Pixmeo SARL, Bernex, Switzerland), with CT-guided region-of-interest (ROI) delineation for quantitative assessment of brain, lung, heart, liver, spleen, kidneys, stomach, and tumor tissues. Standardized uptake values (SUVmax and SUVmean) were derived from these ROIs. Due to the nature of the imaging protocol requiring real-time adjustment of scan parameters based on tumor location, blinding of personnel during image acquisition was not feasible. However, all quantitative analyses (SUV measurements, ROI delineation) were performed using automated software (OsiriX Lite v7.5, Pixmeo SARL, Bernex, Switzerland) with predefined thresholds, minimizing subjective bias. Group assignment was concealed during statistical analysis.

### 4.9. Statistical Methods

Data analysis utilized GraphPad Prism 8 and R software (Version 4.4.1, R Foundation for Statistical Computing, Vienna, Austria). Randomization was implemented for all in vivo experiments. Comparative analyses primarily employed Student’s *t*-tests or two-way ANOVA, with statistical significance defined at *p* < 0.05. Each experimental condition was replicated a minimum of three times unless otherwise specified.

## 5. Conclusions

In conclusion, [^68^Ga]Ga-FL9-7 PARP-1 PET imaging represents a breakthrough in prostate cancer molecular diagnostics, addressing biopsy limitations and enabling precision medicine. With advancing technology and regulatory support, PARP imaging may become standard in 5–10 years. Future research should focus on real-world validation, cost–benefit analysis, and diagnostic impact assessment to maximize clinical potential.

## Data Availability

Data presented in this study are contained within the article.

## Supplementary Materials

The following supporting information can be downloaded at https://www.mdpi.com/article/10.3390/ph19071020/s1: Figure S1: ^1^H-NMR spectrum of compound FL9-3; Figure S2: ^1^H-NMR spectrum of compound FL9-4; Figure S3: ^1^H-NMR spectrum of compound FL9-5; Figure S4: ^13^C-NMR spectrum of compound FL9-5; Figure S5: MS spectrum of compound FL9-3; Figure S6: MS spectrum of compound FL9-4; Figure S7: MS spectrum of compound FL9-5; Figure S8: MS spectrum of compound FL9-6; Figure S9: MS spectrum of compound FL9-7; Figure S10: HPLC analysis of FL9-7; Figure S11: HPLC analysis of ^nat^Ga-FL9-7; Figure S12: Purity of ^68^Ga-FL9-7 by Radio-TLC; Table S1: Molecular docking affinity and key parameters; Table S2: Biodistribution studies of [68Ga]DOTA-FL9-7 in mice bearing 22Rvl xenograft at 1 h, 2 h and 3 h time points after intravenous injection (*n* = 3).
