# Supplementary material for: PET Imaging of New Target PARP in Prostate Cancer
Source: Pharmaceuticals (Basel). 2026 Jun 30;19(7):1020. doi: 10.3390/ph19071020 (PMC13414534; doi:10.3390/ph19071020)
Supplement: Supplementary file 1 [file pharmaceuticals-19-01020-s001.zip › pharmaceuticals-4356017-supplementary.pdf]

Article

PET imaging of new target PARP in prostate cancer

Zhao Yang[1,†], Wei Wang[2,†], Xuanyi Dai[3], Liya Wei[2], Yanli Li[2], Wenfeng Gou[2,*] and Feifei Xu[2,*]

[1] Department of Molecular Imaging and Nuclear Medicine, Tianjin Medical University Cancer Institute and Hospital, National Clinical Research Center for Cancer, Key Laboratory of Cancer Prevention and Therapy, Tianjin's Clinical Research Center for China, Tianjin 300060, China

[2] State Key Laboratory of Advanced Medical Materials and Devices, Tianjin Key Laboratory of Radiation Medicine and Molecular Nuclear Medicine, Tianjin Institutes of Health Science, Institute of Radiation Medicine, Chinese Academy of Medical Sciences & Peking Union Medical College, Tianjin, 300192, China

[3] Tianjin Farragut School, Tianjin 300074, China

† These authors have contributed equally to this work and share first authorship

* Correspondence: gouwenfeng@irm-cams.ac.cn (Wenfeng Gou); xufeifei@irm-cams.ac.cn (Feifei Xu)

Experimental Section

(1-(4-(4,4,5,5-Tetramethyl-1,3,2-dioxaborolan-2-yl)benzyl)piperidin-4-yl)carbamic acid tert-butyl ester (FL9-3)

2.00 g **4-bromomethylbenzoic acid pinacol ester (FL9-1)** (6.60 mmol, 1.0 eq), 1.33 g **4-tert-butoxycarbonylaminopiperidine (FL9-2)** (6.60 mmol, 1.0 eq), and 1.94 g K_2CO_3 (13.86 mmol, 2.1 eq) were dissolved in 40 mL of anhydrous acetonitrile in a single-neck flask. The mixture was stirred at room temperature for 6 h. After monitoring reaction completion via iodine fumigation and thin-layer chromatography (PE: EA = 3:1), ethyl acetate was added to the reaction mixture to dissolve the product. Inorganic salt impurities were removed by vacuum filtration. The filtrate was rotary evaporated under reduced pressure to yield a relatively pure white solid (2.72 g, 98.91%). The crude product required no further purification and was directly used in the subsequent reaction. ^{1}H NMR (400 MHz, DMSO-d_6) δ 7.62 (d, *J* = 7.9 Hz, 2H), 7.29 (d, *J* =7.9 Hz, 2H), 6.77 (d, *J* = 7.9 Hz, 1H), 3.42 (s, 2H), 3.27 – 3.15 (m, 1H), 2.70 (d, *J* = 11.5 Hz, 2H), 1.92 (t, *J* = 10.9 Hz, 2H), 1.64 (d, *J* = 11.1 Hz, 2H), 1.39 – 1.37 (m, 2H), 1.36 (s, 9H), 1.28 (s, 12H). MS-ESI(+) Calcd for $C_{23}H_{37}BN_2O_4$: 416.2846, $[M+H]^+$ found: 417.2936.

tert-Butyl 1-(4-(8-fluoro-1-oxo-2,3,4,6-tetrahydro-1*H*-azepino[5,4,3-*cd*]indol-5-yl)benzyl)piperidin-4-yl)carbamate (FL9-4)

Weigh 1.36 g of the **Rucaparib intermediate (P1)** (4.71 mmol, 1.0 eq), 2.16 g of **FL9-3** (5.18 mmol, 1.1 eq), and 1.00 g of Na_2CO_3 (9.42 mmol, 2.0 eq) into a three-neck flask. Add 40 mL of a DIOX/H_2O mixed solution (DIOX:H_2O = 6:1) to dissolve the solids. Purge the reaction system three times with an argon balloon. Then add 172 mg of Pd(dppf)Cl_2 (0.23 mmol, 0.05 eq) to the reaction system and repeated the purging three times. The mixture was refluxed and stirred at 90°C for 4 h. Monitored by TLC (DCM:MeOH = 20:1) until complete conversion. Add ethyl acetate and water to the reaction mixture. Extract the aqueous phase three times using a separatory funnel. Combine organic layers, wash three times with saturated NaCl aqueous solution, dry over anhydrous Na_2SO_4, filter, and rotary evaporate under reduced pressure to remove the solvent. Crude product obtained. Purification by silica gel column chromatography (DCM:MeOH = 30:1) yielded a pure yellow solid (2.04 g, 87.93%). ^{1}H NMR (400 MHz, DMSO-d_6) δ 11.68 (s, 1H), 8.27 (t, *J* = 5.7 Hz, 1H), 7.58 (d, *J* = 8.2 Hz, 2H), 7.42 (dd, *J* = 10.9, 2.4 Hz, 3H), 7.32 (dd, *J* = 9.1, 2.4 Hz, 1H), 6.79 (d, *J* = 7.8 Hz, 1H), 3.47 (s, 2H), 3.43 – 3.36 (m, 2H), 3.29 – 3.18 (m, 1H), 3.08 – 3.00 (m, 2H), 2.77 (d, *J* = 11.1 Hz, 2H), 1.96 (t, *J* = 11.0 Hz, 2H), 1.68 (d, *J* = 10.7 Hz, 2H), 1.46 – 1.38 (m, 2H), 1.37 (s, 9H). MS-ESI(+) Calcd for $C_{28}H_{33}FN_4O_3$: 492.2537, $[M+H]^+$ found: 493.2637.

5-(4-((4-Aminopiperidin-1-yl)methyl)phenyl)-8-fluoro-2,3,4,6-tetrahydro-1*H*-azepin[5,4,3-*cd*]indol-1-one (FL9-5)

Weigh 817 mg of **FL9-4** (0.27 mmol, 1.66 eq) into a single-neck flask. Add 9 mL of TFA/DCM mixed solvent (TFA:DCM = 1:3) to the reaction system. Stir the reaction mixture at room temperature for 1 hour. Monitor the reaction by TLC until complete conversion (DCM:MeOH = 10:1). Remove the solvent by vacuum distillation. Add anhydrous methanol and acetonitrile, and perform three rounds of

rotary evaporation to remove residual trifluoroacetic acid. Adjust the reaction mixture to alkaline with saturated $NaHCO_3$ aqueous solution and concentrate under reduced pressure to obtain the crude product. Purify by silica gel column chromatography (DCM:MeOH = 5:1) to yield a pure yellow solid (643 mg, 98.79%). ^{1}H NMR (400 MHz, DMSO-d_6) δ 11.87 (s, 1H), 8.30 (t, J = 5.7 Hz, 1H), 7.61 (d, J = 7.7 Hz, 2H), 7.42 (dd, J = 11.0, 2.4 Hz, 3H), 7.35 (dd, J = 9.2, 2.4 Hz, 1H), 3.53 (s, 2H), 3.40 – 3.28 (m, 2H), 3.13 – 3.03 (m, 2H), 3.02 – 2.94 (m, 1H), 2.94 – 2.77 (m, 2H), 2.17 – 1.96 (m, 2H), 1.94 – 1.82 (m, 2H), 1.66 – 1.47 (m, 2H), 1.22 (s, 2H). MS-ESI(+) Calcd for $C_{23}H_{25}FN_4O$: 392.2012, $[M+H]^+$ found: 393.2089.

(S)-2,2',2''-(10-(2-((1-(4-(8-fluoro-1-oxo-2,3,4,6-tetrahydro-1*H*-azepino[5,4,3-*cd*]indol-5-yl)benzyl)piperidin-4-yl)amino)-2-oxoethyl)-1,4,7,10-tetraazacyclododecane-1,4,7-triyl)triacetic acid tri-tert-butyl ester (FL9-6)

Weigh 150 mg of **FL9-6** (0.38 mmol, 1.0 eq), 224 mg of **1,4,7,10-tetrazacyclododecane-1,4,7,10-tetraacetic acid tri-tert-butyl ester (DOTA Ester)** (0.38 mmol, 1.0 eq), and 177 mg of HATU (0.46 mmol, 1.2 eq) into a single-mouth reaction flask. Add 5 mL of DMF to dissolve the solids, then slowly add 1.5 mL of 0.1 M HCl solution dropwise to adjust the pH to 3.0. 1.0 eq), and 177 mg HATU (0.46 mmol, 1.2 eq) into a single-neck flask. Add 5 mL DMF to dissolve the solids, then slowly add 99 mg diluted DIPEA (0.76 mmol, 2.0 eq) to the reaction mixture. Stir the mixture at room temperature for 3 h. Monitoring by TLC indicated complete conversion (DCM:MeOH = 10:1). Ethyl acetate and water were added to the reaction mixture. The aqueous phase was extracted three times using a separatory funnel. The organic phases were combined, washed three times with saturated NaCl aqueous solution, dried over anhydrous Na_2SO_4, filtered, and the solvent was removed under reduced pressure to afford the crude product. Purification by silica gel column chromatography (DCM:MeOH = 80:1) yielded a pure pale yellow solid (313 mg, 86.84%). MS-ESI(+) Calcd for $C_{51}H_{75}FN_8O_8$: 946.5692, $[M+H]^+$ found: 947.5792, $[M+Na]^+$ found: 969.5607.

2,2',2''-(10-(2-((1-(4-(8-fluoro-1-oxo-2,3,4,6-tetrahydro-1*H*-azepino[5,4,3-*cd*]indol

-5-yl)benzyl)piperidin-4-yl)amino)-2-oxoethyl)-1,4,7,10-tetraazacyclododecane-1,4,7-triyl)triacetic acid (FL9-7)

Weigh 310 mg of FL0-5 (0.33 mmol, 1.0 eq) into a single-neck flask. Dissolve in 3 mL of TFA/DCM mixture (TFA:DCM = 2:1) and stir at room temperature for 24 h. Monitor the reaction by TLC until complete conversion (DCM:MeOH = 5:1). Remove the solvent by vacuum distillation. Add anhydrous methanol and acetonitrile, and perform three rounds of rotary evaporation to remove residual trifluoroacetic acid. Dissolve the reaction mixture in a small amount of methanol, then add 10-fold volume of methyl tert-butyl ether to precipitate the product. Filter and collect the cake, dry at 40°C for 6 h to obtain a pure pale yellow solid (200 mg, 78.78%). MS-ESI(+) Calcd for $C_{39}H_{51}FN_8O_8$: 778.3814, $[M+H]^+$ found: 779.3879. HPLC purity: 97.01%.

Supporting information

1 NMR spectra of intermediates and target compounds

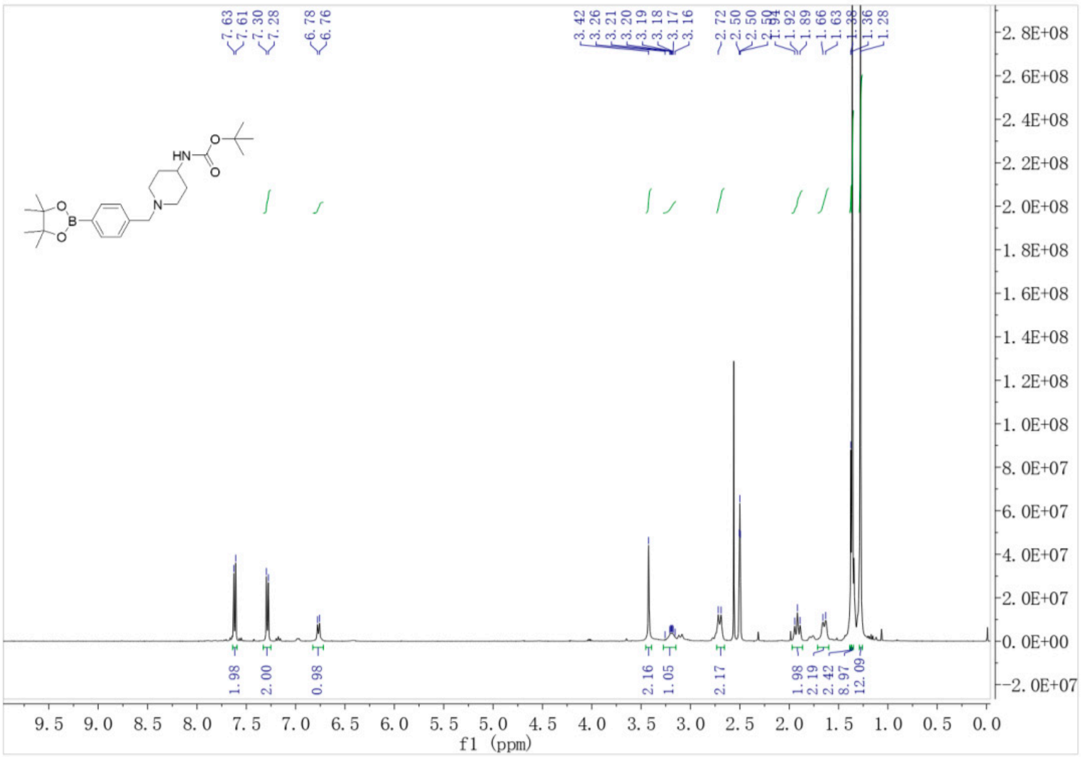

Figure S1 ^{1}H-NMR spectrum of compound **FL9-3**

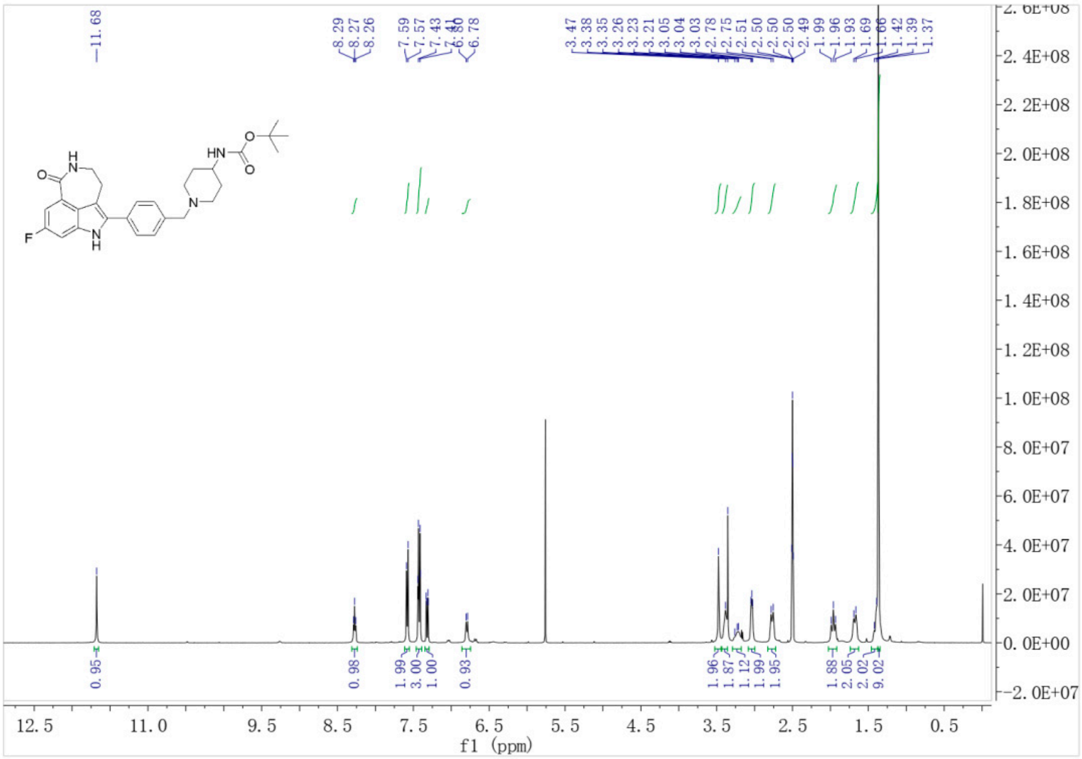

Figure S2 ^{1}H-NMR spectrum of compound **FL9-4**

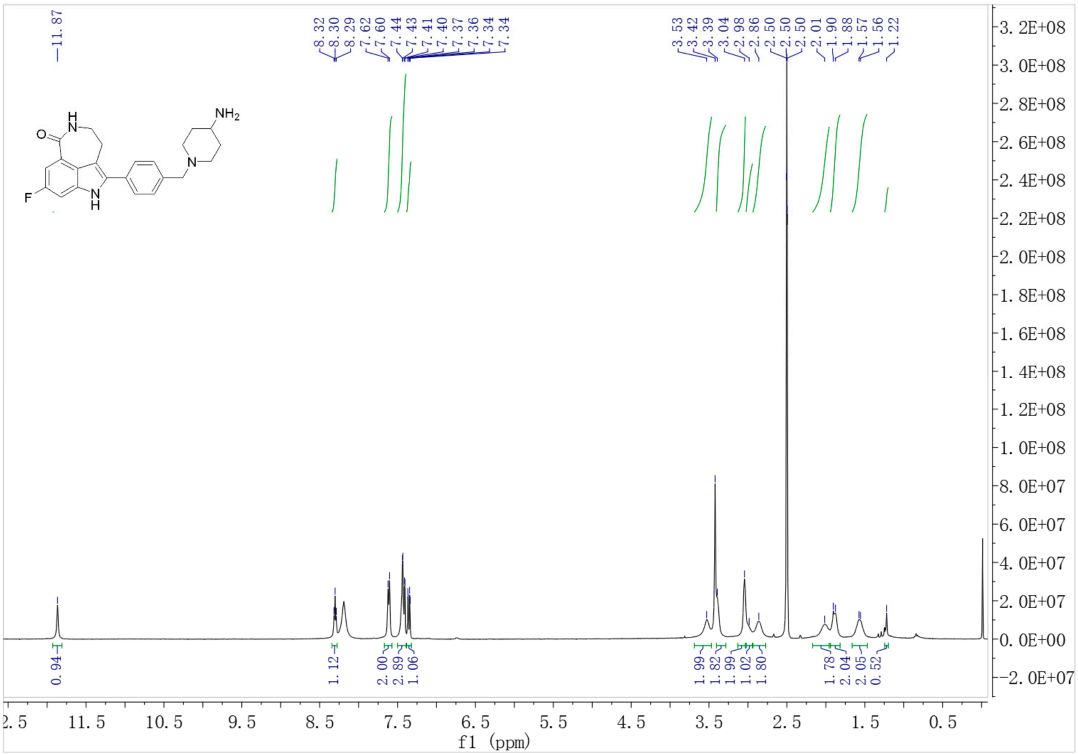

Figure S3 ^{1}H-NMR spectrum of compound **FL9-5**

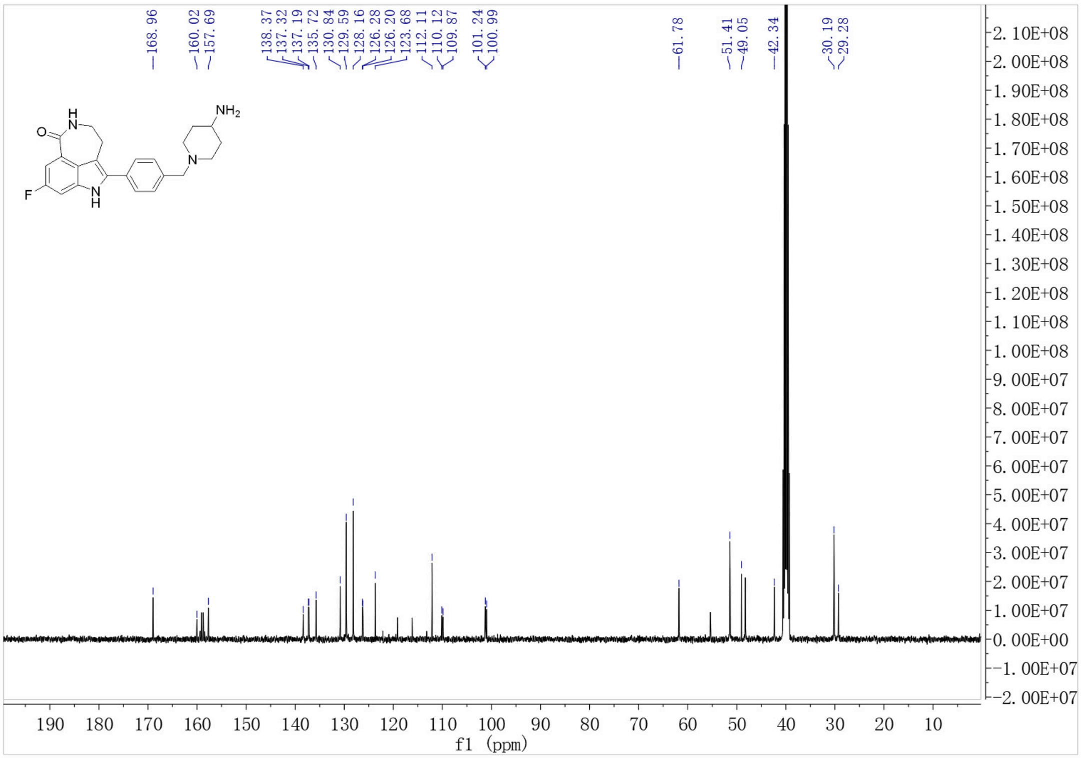

Figure S4 ^{13}C-NMR spectrum of compound **FL9-5**

2 Mass Spectrometry of Intermediates and Target Compounds

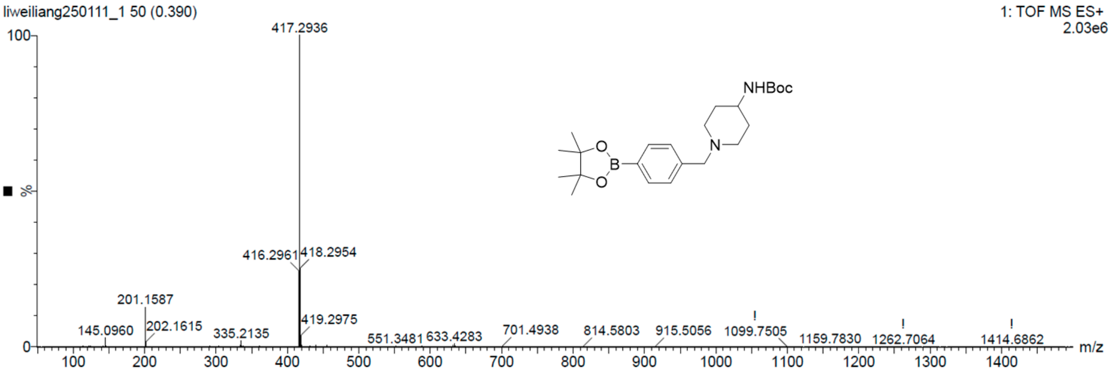

Figure S5 MS spectrum of compound **FL9-3**

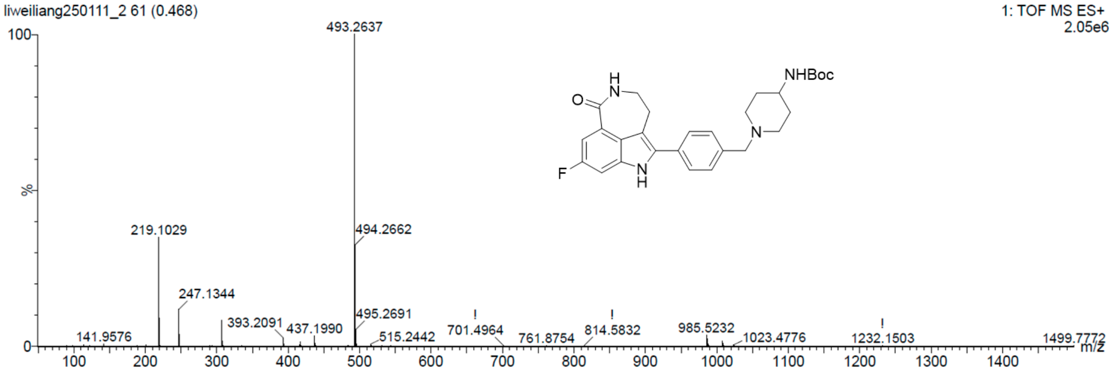

Figure S6 MS spectrum of compound **FL9-4**

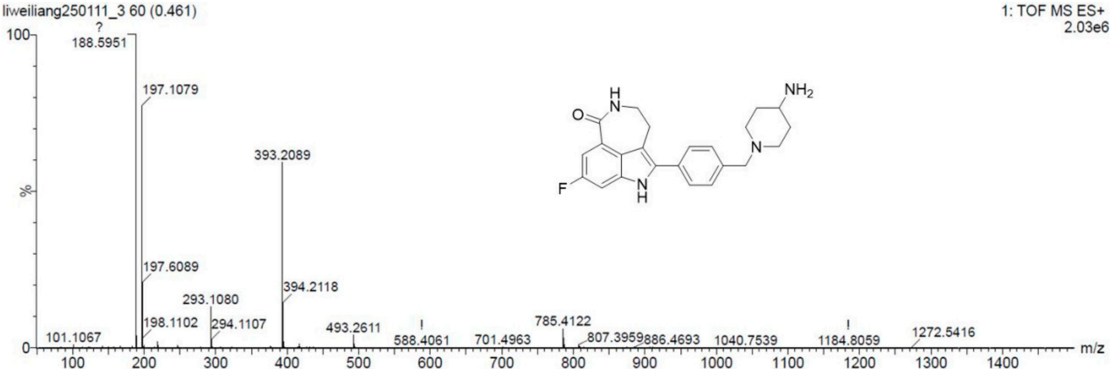

Figure S7 MS spectrum of compound **FL9-5**

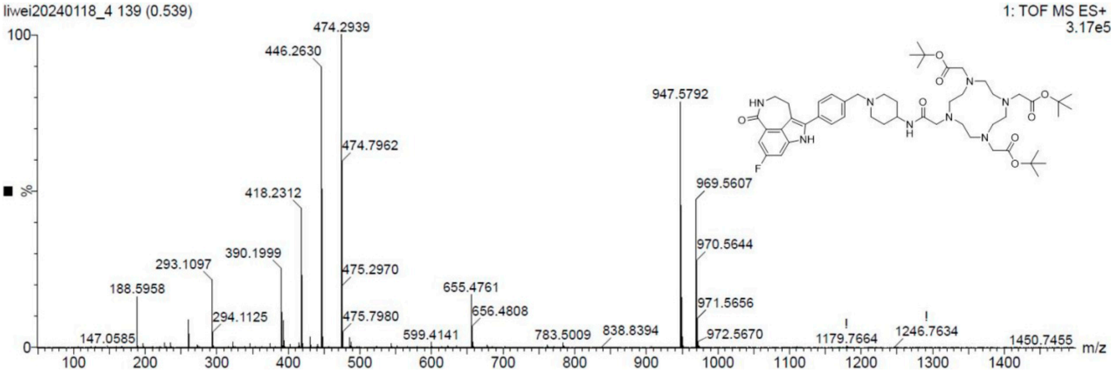

Figure S8 MS spectrum of compound **FL9-6**

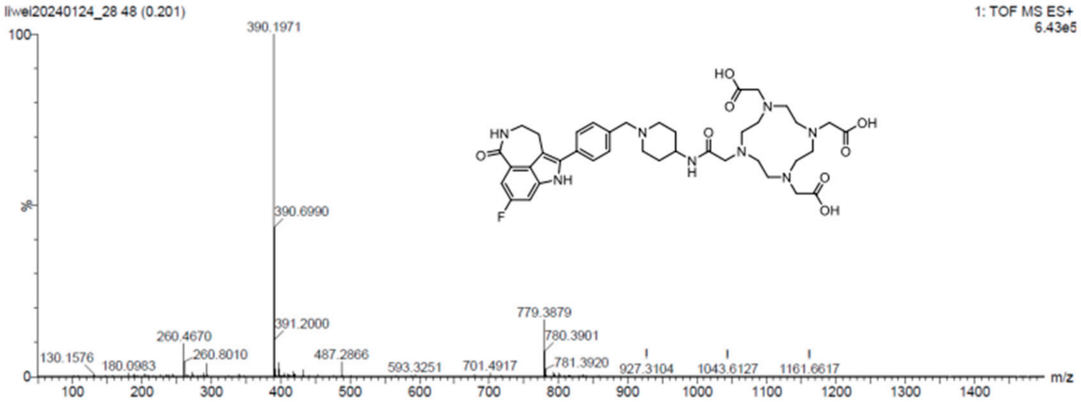

Figure S9 MS spectrum of compound **FL9-7**

<色谱图>

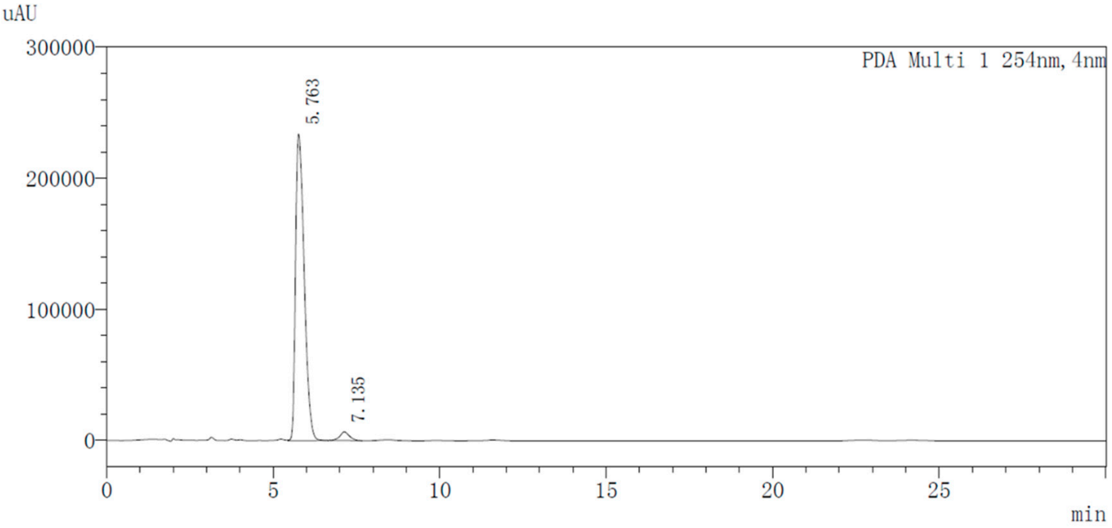

<峰表>

PDA Ch1 254nm

峰号	保留时间	面积	面积%	高度	理论塔板数(USP)	分离度(USP)	拖尾因子
1	5.763	4231717	97.008	233936	2331	--	1.571
2	7.135	130521	2.992	6655	3161	2.785	1.046
总计		4362238	100.000	240590			

Figure S10 HPLC analysis of **FL9-7**

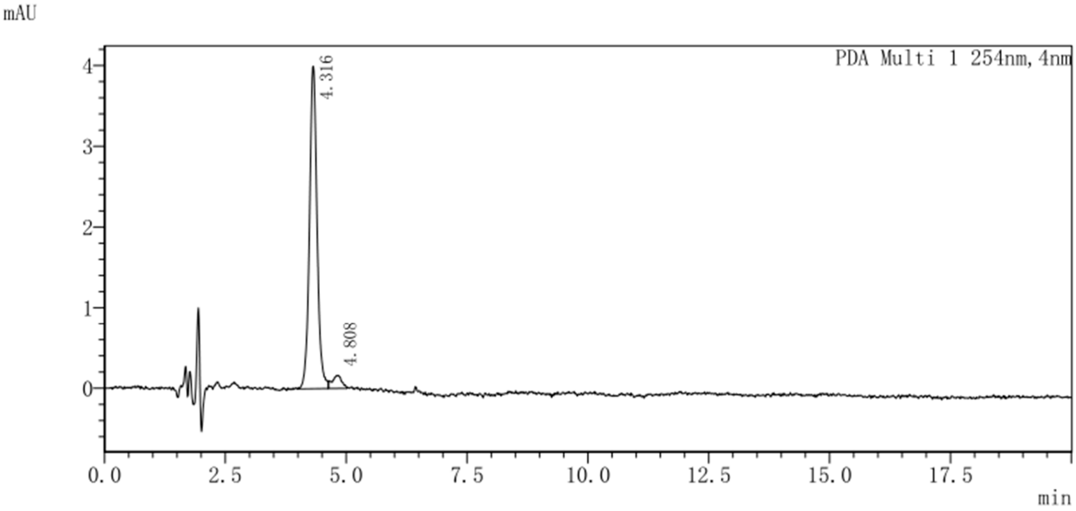

PDA Ch1 254nm

峰号	保留时间	面积	面积%	高度	理论塔板数(USP)	分离度(USP)	拖尾因子
1	4.316	43512	95.056	3998	3574	--	1.072
2	4.808	2263	4.944	163	2459	1.456	--
总计		45776	100.000	4160			

Figure S11 HPLC analysis of **^{nat}Ga-FL9-7**

Reg	(mm) Start	(mm) Stop	(mm) Centroid	RF	Region Counts	Region CPM	% of Total	% of ROI
Rgn 1	-0.4	21.8	10.9	0.015	2177.0	2177.0	1.75	1.78
Rgn 2	40.3	80.2	59.5	0.824	119921.0	119921.0	96.13	98.22
2 Peaks					122098.0	122098.0	97.87	100.00

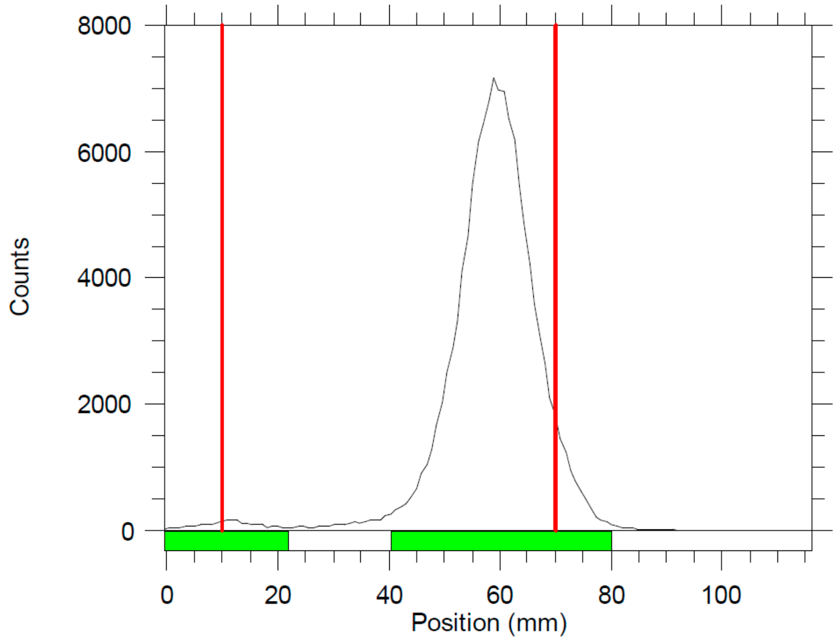

Figure S12 Purity of ^{68}**Ga-FL9-7** by Radio-TLC

Table S1, Molecular docking affinity and key parameters

Title	glide gscore	docking score	glide emodel	XP GScore	XP HBond	IFDScore
FL1	-11.474	-10.29	-123.667	-11.474	-3.581	-469.19
FL1	-12.269	-11.085	-113.733	-12.269	-1.89	-468.65
FL1	-11.6	-10.416	-119.264	-11.6	-1.993	-468.46
FL1	-11.17	-9.986	-110.478	-11.17	-2.701	-467.9
FL1	-10.971	-9.787	-127.519	-10.971	-1.53	-467.57
FL1	-9.989	-8.805	-127.183	-9.989	-2.25	-466.24
FL1	-11.546	-11.345	-118.144	-11.546	-1.479	-468.48
FL1	-9.571	-9.37	-132.376	-9.571	-2.763	-466.03
FL1	-9.456	-9.255	-97.019	-9.456	-1.485	-465.1
FL1	-10.074	-7.392	-111.447	-10.074	-2.413	-466.05
FL1	-9.344	-6.662	-110.841	-9.344	-1.657	-465.32
FL1	-8.736	-6.054	-100.394	-8.736	-1.064	-465.13
FL1	-8.54	-5.858	-111.313	-8.54	-1.05	-464.98
FL1	-8.71	-6.028	-110.971	-8.71	-1.942	-464.9
FL1	-8.227	-5.545	-104.061	-8.227	-0.876	-464.48
FL1	-8.293	-5.611	-113.02	-8.293	-2.173	-464.27
FL1	-8.087	-5.405	-101.542	-8.087	-1.963	-464.18
FL1	-8.069	-5.387	-103.55	-8.069	-1.184	-464.15
FL1	-8.108	-5.426	-97.553	-8.108	-0.755	-463.85
FL1	-7.531	-4.849	-89.979	-7.531	-1.35	-463.78
FL1	-7.712	-5.031	-95.964	-7.712	-1.252	-463.75
FL1	-8.261	-5.58	-97.405	-8.261	-1.703	-463.75
FL1	-8.347	-5.665	-103.191	-8.347	-1.588	-463.59
FL1	-7.314	-4.632	-114.689	-7.314	-1.575	-462.61
FL1	-6.174	-3.492	-98.004	-6.174	-0.784	-461.74
FL1	-5.487	-2.805	-65.713	-5.487	-0.361	-461.11
FL1	-12.101	-10.942	-123.62	-12.101	-2.988	-464.5
FL1	-10.443	-9.285	-141.738	-10.443	-1.754	-462.52
FL1	-9.416	-8.258	-127.941	-9.416	-2.855	-460.68
FL10	-10.398	-9.24	-122.589	-10.398	-2.487	-466.83
FL10	-10.401	-9.242	-120.728	-10.401	-3.51	-466.76
FL10	-10.625	-7.937	-130.982	-10.625	-1.408	-467.11
FL10	-11.2	-8.512	-99.861	-11.2	-1.05	-466.92
FL10	-10.443	-7.754	-117.309	-10.443	-1.129	-466.59
FL10	-10.163	-7.475	-116.158	-10.163	-2.192	-466.26
FL10	-10.26	-7.571	-121.185	-10.26	-2.441	-466.22
FL10	-9.184	-6.496	-127.638	-9.184	-1.384	-465.81
FL10	-10.076	-7.388	-105.968	-10.076	-1.763	-465.77
FL10	-9.064	-6.375	-107.517	-9.064	-1.221	-465.69
FL10	-9.32	-6.632	-115.542	-9.32	-1.091	-465.66
FL10	-8.704	-6.016	-100.347	-8.704	-1.704	-465.43

FL10	-9.264	-6.575	-102.481	-9.264	-1.411	-464.89
FL10	-8.161	-5.473	-110.627	-8.161	-1.33	-464.72
FL10	-7.821	-5.133	-84.245	-7.821	-0.544	-464.52
FL10	-8.658	-5.969	-97.178	-8.658	-1.978	-464.41
FL10	-8.761	-6.073	-85.147	-8.761	-1.495	-464.38
FL10	-7.904	-5.216	-94.727	-7.904	-1.429	-463.82
FL10	-7.874	-5.186	-98.497	-7.874	-1.105	-463.62
FL10	-7.98	-5.292	-96.244	-7.98	-1.071	-463.26
FL10	-10.745	-10.539	-113.888	-10.745	-2.589	-467.04
FL10	-9.478	-9.272	-114.229	-9.478	-1.313	-465.7
FL10	-9.199	-8.993	-107.534	-9.199	-2.783	-465.01
FL10	-8.988	-8.782	-89.618	-8.988	-1.151	-464.91
FL10	-9.165	-8.959	-113.029	-9.165	-2.022	-464.64
FL10	-9.168	-8.962	-112.907	-9.168	-2.631	-464.43
FL10	-8.864	-8.658	-107.285	-8.864	-2.172	-464.05
FL10	-8.805	-8.599	-101.783	-8.805	-0.766	-464
FL10	-7.81	-7.604	-93.806	-7.81	-1.625	-463.8
FL10	-8.504	-8.298	-102.562	-8.504	-1.224	-463.68
FL10	-7.65	-7.444	-93.088	-7.65	-1.459	-463.44
FL10	-7.447	-7.241	-85.9	-7.447	-1.636	-463.28
FL10	-7.33	-7.124	-102.498	-7.33	-0.7	-462.79
FL10	-9.726	-8.567	-112.746	-9.726	-1.561	-466.05
FL10	-10.108	-8.949	-112.049	-10.108	-0.833	-465.62
FL10	-9.832	-8.674	-118.756	-9.832	-2.45	-465.01
FL10	-9.864	-8.706	-119.205	-9.864	-3.454	-464.85
FL10	-8.631	-7.473	-115.007	-8.631	-1.176	-464.35
FL2	-11.645	-8.926	-105.81	-11.645	-2.702	-468.05
FL2	-11.045	-8.326	-97.366	-11.045	-1.95	-467.85
FL2	-11.911	-9.192	-90.752	-11.911	-3.389	-467.7
FL2	-12.542	-11.709	-144.831	-12.542	-3.06	-468.65
FL2	-12.126	-11.293	-135.942	-12.126	-2.488	-467.51
FL2	-9.377	-8.544	-114.152	-9.377	-2.089	-464.21
FL2	-10.437	-8.894	-136.834	-10.437	-1.683	-465.84
FL2	-9.442	-7.898	-120.316	-9.442	-1.251	-464.65
FL2	-9.569	-8.025	-108.595	-9.569	-2.057	-464.58
FL2	-10.1	-8.556	-115.978	-10.1	-2.57	-464.5
FL2	-9.122	-7.578	-125.966	-9.122	-1.996	-464.2
FL2	-9.398	-7.854	-114.168	-9.398	-1.145	-464.04
FL2	-9.518	-7.974	-122.037	-9.518	-2.75	-463.77
FL2	-9.235	-7.691	-105.436	-9.235	-1.81	-463.73
FL2	-8.958	-7.415	-126.541	-8.958	-2.262	-463.55
FL2	-9.413	-7.869	-112.2	-9.413	-2.128	-463.5
FL2	-8.908	-7.364	-112.984	-8.908	-1.468	-463.36
FL2	-8.809	-7.265	-114.511	-8.809	-1.2	-463.3

FL2	-9.137	-7.593	-115.153	-9.137	-1.44	-463.27
FL2	-8.377	-6.833	-112.797	-8.377	-1.295	-462.56
FL2	-6.876	-5.332	-104.872	-6.876	-2.217	-461.37
FL3	-11.491	-10.334	-114.898	-11.491	-1.422	-466.71
FL3	-11.299	-10.142	-124.753	-11.299	-4.084	-466.71
FL3	-11.63	-10.473	-126.043	-11.63	-2.682	-466.65
FL3	-11.503	-10.346	-102.932	-11.503	-1.944	-466.53
FL3	-10.545	-9.388	-129.673	-10.545	-4.909	-466.25
FL3	-11.578	-10.421	-113.136	-11.578	-1.285	-466.19
FL3	-11.414	-10.257	-121.692	-11.414	-2.689	-465.9
FL3	-9.792	-8.635	-122.732	-9.792	-2.125	-465.64
FL3	-10.449	-9.292	-132.116	-10.449	-3.015	-465.57
FL3	-10.612	-9.453	-127.697	-10.612	-1.379	-465.38
FL3	-9.753	-8.595	-129.822	-9.753	-2.331	-464.94
FL3	-9.884	-8.726	-138.32	-9.884	-1.905	-463.6
FL3	-9.399	-8.241	-126.1	-9.399	-1.884	-463.58
FL3	-8.83	-7.672	-134.414	-8.83	-2.087	-463.24
FL3	-7.727	-6.568	-103.42	-7.727	-0.665	-462.74
FL3	-8.088	-6.93	-101.475	-8.088	-0.97	-462.68
FL3	-7.871	-6.713	-103.618	-7.871	-1.782	-461.91
FL3	-6.487	-5.329	-88.913	-6.487	-2.02	-460.55
FL3	-9.548	-6.861	-114.031	-9.548	-1.424	-465.3
FL3	-7.649	-4.962	-90.622	-7.649	-1.353	-462.23
FL3	-9.21	-9.004	-116.697	-9.21	-2.102	-465.06
FL3	-8.882	-8.676	-99.421	-8.882	-1.865	-464.2
FL3	-9.256	-9.05	-115.491	-9.256	-2.109	-464.19
FL3	-8.834	-8.627	-104.393	-8.834	-1.208	-464.18
FL3	-7.852	-7.646	-107.051	-7.852	-1.317	-462.29
FL3	-7.273	-7.067	-107.209	-7.273	-1.263	-462.11
FL4	-11.528	-10.371	-107.949	-11.528	-1.255	-466.67
FL4	-11.564	-10.407	-96.458	-11.564	-1.91	-466.65
FL4	-11.103	-9.946	-109.012	-11.103	-0.934	-466.55
FL4	-11.659	-10.502	-109.703	-11.659	-1.846	-466.12
FL4	-11.355	-10.198	-107.141	-11.355	-2.44	-466.04
FL4	-9.985	-8.828	-119.582	-9.985	-1.96	-465.26
FL4	-9.045	-7.888	-109.959	-9.045	-2.111	-464.6
FL4	-9.839	-8.682	-117.206	-9.839	-2.164	-464.05
FL4	-8.402	-7.245	-113.67	-8.402	-1.156	-462.96
FL4	-9.429	-6.742	-125.581	-9.429	-1.381	-466.6
FL4	-9.469	-6.782	-120.109	-9.469	-1.552	-466.47
FL4	-8.397	-5.71	-107.589	-8.397	-2.019	-466.04
FL4	-9.363	-6.676	-106.959	-9.363	-1.42	-465.74
FL4	-8.946	-6.259	-106.512	-8.946	-1.509	-465.34
FL4	-6.733	-4.046	-91.206	-6.733	-1.03	-462.96

FL4	-10.247	-10.041	-117.38	-10.247	-2.813	-466.12
FL4	-9.947	-9.741	-123.317	-9.947	-2.386	-466.06
FL4	-9.899	-9.693	-121.981	-9.899	-3.82	-465.73
FL4	-10.547	-10.341	-125.242	-10.547	-1.81	-465.6
FL4	-10.295	-10.089	-109.651	-10.295	-3.268	-465.45
FL4	-9.431	-9.224	-102.259	-9.431	-1.409	-464.7
FL4	-9.222	-9.015	-119.43	-9.222	-2.327	-464.65
FL4	-9.302	-9.096	-115.005	-9.302	-2.725	-464.63
FL4	-9.253	-9.047	-108.621	-9.253	-1.968	-464.43
FL4	-7.695	-7.489	-94.678	-7.695	-1.224	-463.22
FL4	-8.169	-7.963	-98.979	-8.169	-0.958	-463.1
FL4	-7.547	-7.34	-94.252	-7.547	-0.943	-463
FL4	-7.031	-6.825	-92.067	-7.031	-0.932	-462.55
FL4	-6.934	-6.728	-96.913	-6.934	-1.117	-461.85
FL5	-13.401	-12.242	-104.955	-13.401	-4.113	-469.96
FL5	-13.962	-12.804	-123.581	-13.962	-2.242	-469.02
FL5	-13.461	-12.302	-119.179	-13.461	-1.575	-468.89
FL5	-12.957	-11.799	-130.441	-12.957	-1.96	-468.43
FL5	-12.637	-11.478	-126.22	-12.637	-1.995	-468.34
FL5	-12.632	-11.473	-124.089	-12.632	-1.961	-468.08
FL5	-12.013	-10.854	-131.456	-12.013	-1.359	-467.67
FL5	-11.866	-10.707	-112.331	-11.866	-1.514	-467.45
FL5	-11.694	-10.536	-133.381	-11.694	-3.704	-466.97
FL5	-11.213	-10.055	-110.534	-11.213	-1.33	-466.28
FL5	-9.097	-7.938	-102.498	-9.097	-2.627	-465.19
FL5	-11.562	-8.874	-96.052	-11.562	-1.4	-467.59
FL5	-10.006	-7.318	-85.273	-10.006	-0.902	-466.92
FL5	-9.786	-7.098	-81.82	-9.786	-1.323	-466.39
FL5	-8.589	-5.901	-112.266	-8.589	-2.269	-465.7
FL5	-8.787	-6.099	-114.53	-8.787	-1.904	-465.54
FL5	-8.84	-6.152	-91.525	-8.84	-2.604	-465.06
FL5	-8.188	-5.5	-102.801	-8.188	-0.93	-464.75
FL5	-7.778	-5.09	-86.041	-7.778	-1.512	-464.05
FL5	-7.734	-5.046	-93.353	-7.734	-1.77	-463.67
FL5	-7.796	-5.108	-100.109	-7.796	-1.561	-463.62
FL5	-7.414	-4.726	-88.503	-7.414	-1.153	-463.34
FL5	-6.73	-4.041	-88.287	-6.73	-0.493	-463.15
FL5	-6.444	-3.755	-90.828	-6.444	-1.204	-462.81
FL5	-6.81	-4.122	-88.369	-6.81	-1.296	-462.67
FL5	-10.407	-10.201	-115.77	-10.407	-2.581	-467.09
FL5	-10.559	-10.353	-106.429	-10.559	-3.254	-466.6
FL5	-10.968	-10.762	-123.615	-10.968	-2.454	-466.21
FL5	-9.779	-9.573	-109.757	-9.779	-3.23	-466.02
FL5	-8.989	-8.783	-111.862	-8.989	-1.022	-465.21

FL5	-9.361	-9.155	-100.867	-9.361	-2.039	-465
FL5	-8.719	-8.513	-108.121	-8.719	-1.196	-464.57
FL5	-8.825	-8.619	-118.269	-8.825	-2.035	-464.52
FL5	-8.138	-7.932	-102.144	-8.138	-1.151	-463.58
FL5	-9.818	-8.66	-126.933	-9.818	-2.289	-464.41
FL5	-9.086	-7.927	-125.078	-9.086	-1.356	-463.71
FL5	-9.244	-8.086	-124.75	-9.244	-1.898	-463.1
FL5	-8.312	-7.154	-118.532	-8.312	-1.488	-463
FL5	-9.296	-8.137	-121.412	-9.296	-1.769	-462.8
FL5	-8.119	-6.96	-117.155	-8.119	-0.787	-462.43
FL5	-8.335	-7.177	-113.346	-8.335	-2.416	-462.17
FL5	-8.003	-6.845	-121.342	-8.003	-1.529	-462.13
FL5	-8.139	-6.98	-111.93	-8.139	-2.066	-461.98
FL5	-8.446	-7.287	-117.991	-8.446	-1.118	-461.95
FL5	-8.198	-7.04	-115.666	-8.198	-0.7	-461.86
FL5	-8.069	-6.911	-107.825	-8.069	-1.33	-461.78
FL5	-7.582	-6.424	-114.966	-7.582	-1.329	-461.32
FL5	-7.928	-6.77	-93.872	-7.928	-1.36	-461.24
FL5	-6.85	-5.691	-111.188	-6.85	-1.302	-461.1
FL5	-7.125	-5.966	-107.595	-7.125	-0.185	-461.08
FL5	-6.965	-5.807	-94.841	-6.965	-1.348	-460.16
FL6	-10.877	-9.692	-110.82	-10.877	-1.27	-466.88
FL6	-11.309	-10.15	-141.718	-11.309	-1.928	-466.36
FL6	-10.499	-9.34	-124.002	-10.499	-1.372	-466.01
FL6	-8.724	-7.566	-115.443	-8.724	-1.199	-464.4
FL6	-8.56	-7.401	-125.03	-8.56	-1.924	-464.18
FL6	-8.549	-7.391	-111.305	-8.549	-1.81	-463.65
FL6	-9.085	-7.927	-116.93	-9.085	-1.589	-463.61
FL6	-9.016	-7.857	-125.937	-9.016	-0.924	-463.4
FL6	-8.062	-6.904	-112.793	-8.062	-0.417	-463.14
FL6	-8.393	-7.234	-116.788	-8.393	-1.007	-462.62
FL6	-7.428	-6.27	-104.391	-7.428	-1.142	-462.36
FL6	-9.591	-6.909	-98.812	-9.591	-1.551	-465.84
FL6	-9.489	-6.807	-100.794	-9.489	-0.7	-465.21
FL6	-7.662	-4.98	-109.767	-7.662	-1.553	-463.93
FL6	-8.361	-5.679	-93.102	-8.361	-1.874	-463.5
FL6	-7.099	-4.417	-94.911	-7.099	-0.997	-462.73
FL6	-6.521	-3.839	-77.917	-6.521	-0.331	-461.3
FL7	-11.949	-9.442	-124.984	-11.949	-1.33	-469.5
FL7	-12.106	-9.598	-131.097	-12.106	-2.835	-468.87
FL7	-11.861	-9.353	-89.837	-11.861	-1.073	-468.72
FL7	-11.671	-9.163	-116.613	-11.671	-2.197	-468.64
FL7	-10.93	-8.422	-98.782	-10.93	-1.581	-468.4
FL7	-12.047	-9.539	-111.745	-12.047	-3.229	-468.38

FL7	-11.734	-9.227	-119.396	-11.734	-2.52	-468.05
FL7	-11.24	-8.732	-103.825	-11.24	-2.371	-467.55
FL7	-9.833	-7.325	-103.174	-9.833	-1.464	-467.52
FL7	-10.701	-8.193	-102.186	-10.701	-1.934	-467.42
FL7	-9.853	-7.346	-115.069	-9.853	-1.885	-466.83
FL7	-9.627	-7.12	-129.693	-9.627	-1.768	-466.66
FL7	-9.358	-6.85	-98.589	-9.358	-1.623	-466.48
FL7	-9.835	-7.328	-113.667	-9.835	-1.378	-466.14
FL7	-12.132	-12.107	-124.421	-12.132	-1.89	-467.25
FL7	-11.31	-11.285	-114.454	-11.31	-1.81	-466.32
FL7	-12.167	-12.142	-121.178	-12.167	-2.139	-466.22
FL7	-12.319	-12.294	-115.324	-12.319	-2.1	-466.15
FL7	-11.54	-11.515	-123.323	-11.54	-3.282	-465.96
FL7	-11.871	-11.846	-127.469	-11.871	-1.916	-465.59
FL7	-11.234	-11.209	-129.037	-11.234	-4.481	-465.54
FL7	-10.308	-10.284	-116.569	-10.308	-1.273	-465.04
FL7	-11.189	-11.165	-123.472	-11.189	-2.45	-464.89
FL7	-11.142	-11.117	-123.832	-11.142	-2.963	-464.77
FL7	-9.877	-9.853	-106.327	-9.877	-1.78	-464.11
FL7	-10.077	-10.052	-121.298	-10.077	-2.252	-463.9
FL7	-9.914	-9.889	-107.083	-9.914	-2.544	-463.79
FL7	-9.56	-7.412	-114.641	-9.56	-2.697	-466.89
FL7	-10.204	-8.055	-118.048	-10.204	-1.46	-466.51
FL7	-9.997	-7.849	-120.983	-9.997	-3.017	-466.07
FL7	-8.916	-6.767	-105.274	-8.916	-1.829	-465.25
FL7	-9.148	-6.999	-104.04	-9.148	-1.249	-465.18
FL7	-8.657	-6.509	-112.045	-8.657	-3.418	-464.74
FL7	-7.592	-5.443	-103.082	-7.592	-2.127	-463.64
FL7	-7.457	-5.308	-117.41	-7.457	-2.832	-463.38
FL7	-7.179	-5.03	-99.354	-7.179	-1.38	-463.26
FL8	-11.122	-8.402	-127.297	-11.122	-1.855	-466.72
FL8	-11.065	-8.346	-124.485	-11.065	-2.066	-466.63
FL8	-12.101	-9.382	-120.929	-12.101	-2.793	-466.5
FL8	-10.731	-8.012	-97.804	-10.731	-2.107	-464.94
FL8	-9.451	-6.732	-104.653	-9.451	-1.6	-464.63
FL9	-12.853	-11.696	-119.8	-12.853	-2.273	-468.35
FL9	-11.929	-10.772	-102.288	-11.929	-1.76	-467.78
FL9	-11.536	-10.379	-107.635	-11.536	-3.108	-466.73
FL9	-10.552	-9.395	-121.511	-10.552	-2.55	-466.61
FL9	-11.77	-10.613	-86.467	-11.77	-2.583	-466.36
FL9	-8.147	-6.99	-98.104	-8.147	-0.446	-463.17
FL9	-11.239	-11.032	-117.632	-11.239	-1.885	-466.1
FL9	-9.697	-9.491	-128.216	-9.697	-2.04	-464.86
FL9	-8.749	-8.543	-98.658	-8.749	-1.996	-463.33

FL9	-8.819	-8.613	-108.651	-8.819	-2.155	-463.31
FL9	-8.417	-8.211	-103.946	-8.417	-1.568	-462.97
FL9	-9.292	-9.086	-113.172	-9.292	-2.569	-462.97
FL9	-7.898	-7.692	-100.134	-7.898	-2.244	-462.93
FL9	-8.377	-8.171	-84.187	-8.377	-2.825	-462.6
FL9	-5.901	-3.214	-84.916	-5.901	-0.7	-461.65
FL9	-10.198	-9.039	-133.211	-10.198	-3.091	-465.83
FL9	-10.767	-9.609	-115.318	-10.767	-2.187	-465.29
FL9	-9.159	-8	-113.346	-9.159	-0.933	-464.9
FL9	-9.255	-8.096	-128.713	-9.255	-1.93	-464.49
FL9	-9.109	-7.951	-112.439	-9.109	-2.08	-463.99
FL9	-8.24	-7.082	-113.601	-8.24	-1.41	-463.75
FL9	-8.7	-7.542	-116.28	-8.7	-1.535	-463.68
FL9	-8.043	-6.885	-119.339	-8.043	-0.83	-463.67
FL9	-7.907	-6.749	-81.251	-7.907	-1.75	-463.23
FL9	-7.848	-6.689	-106.404	-7.848	-1.562	-462.9
FL9	-7.728	-6.569	-109.367	-7.728	-0.798	-462.28

Table S2, Biodistribution studies of **[^{68}Ga]DOTA-FL9-7** in mice bearing 22Rvl xenograft at 1 h, 2 h and 3 h time points after intravenous injection (n=3)

Time<br>Organ<br>(SUV)	Heart	Liver	Spleen	Lung	Kidney	Stomach	Brain	Tumor
1 h	0.4685	0.5155	0.4883	0.3397	3.5600	0.2579	0.0999	0.2853
2 h	0.2515	0.4550	0.3484	0.1909	3.5500	0.2069	0.0779	0.3384
3 h	0.1603	0.4023	0.2510	0.0955	0.3928	0.1714	0.0449	0.3533
